# Draft genome sequence of freshwater *Roseomonas* sp. strain GC11, isolated from Conservancy Park Lake in Belleview, KY, USA

**DOI:** 10.1128/mra.00462-25

**Published:** 2025-08-11

**Authors:** Kellyn R. Dolezal, Joshua T. Cooper

**Affiliations:** 1 Department of Chemistry & Biochemistry, Northern Kentucky University 3897, Highland Heights, Kentucky, USA; 2 Department of Biological Sciences, Northern Kentucky University 3897, Highland Heights, Kentucky, USA; California State University San Marcos, San Marcos, California, USA

**Keywords:** *Roseomonas*, genome sequence, freshwater lake, genomic characterization

## Abstract

Here we report the draft genome of *Roseomonas* sp. strain GC11 isolated from a freshwater lake, occupying an old gravel quarry, in Belleview, Kentucky. The GC11 genome is 5.25 Mb and contains genes associated with complex carbohydrate degradation and a possible role in nitrite and thiosulfate reduction in aquatic habitats.

## ANNOUNCEMENT

The genus *Roseomonas* is commonly associated with hospital-acquired infections, particularly catheter-related cases (1, 2) and is a recognized human pathogen (3, 4). However, 16S rRNA surveys (5–7) and increasing numbers of environmental isolates (8–11) suggest that *Roseomonas* is more ecologically widespread than previously thought (12).

While characterizing bacterial diversity in urban and human-impacted aquatic habitats, we isolated and sequenced strain GC11 to explore its ecological role and metabolic potential. The genome of GC11 suggests involvement in nitrogen and sulfur cycling and contributes to our understanding of microbial diversity in restored ecosystems. *Roseomonas* sp. strain GC11 was isolated from a surface water grab collected aseptically in a WhirlPak bag containing a small amount of sediment from the shoreline of Conservancy Park Lake in Bellview, Northern Kentucky (38.992717°N, 84.823078°W), in September 2021. The lake occupies a former gravel pit, reclaimed and restored in 2007. The sample was serially diluted (1:10), plated on Reasoner’s 2A agar (RPI), and incubated at 25°C for 48 h. Colonies appeared distinctively pink with runny margins, consistent with previous *Roseomonas* descriptions (13). Genomic DNA was extracted from a single colony grown in Tryptic Soy Broth (RPI) and incubated at 25°C for 48 h. DNA was isolated using the Qiagen UltraClean Microbial DNA Isolation Kit (Qiagen, MD, USA) following the manufacturer’s protocol. DNA concentration was measured using the Qubit 3.0 broad range kit (Invitrogen, Carlsbad, CA), and the sample was submitted for whole-genome sequencing (SeqCenter LLC, PA). Library preparation was performed using the Illumina Nextera XL Kit and sequenced on an Illumina NextSeq 2000 (2 × 151 bp), yielding 1,778,631 paired-end reads.

Reads were analyzed and assembled with KBase v2.3.2 (14). Quality control was performed with FastQC v0.11.9 (15), and the reads were trimmed using Nextera adapters with Trimmomatic v0.36 (16). Genome assembly was performed using SPAdes v3.15.3 (17), and genome completeness (100%) and contamination (0%) were assessed with CheckM v1.0.18 (18). Final annotations were generated with the NCBI Prokaryotic Genome Annotation Pipeline (PGAP) v6.1 (19). Default parameters were used.

The assembly was 5,252,435 bp, across 212 contigs, with a GC% content of 70.72%, and an N_50_ of 53,914 bp. The average coverage was 93.7×, calculated with Qualimap v2.2.1 (20). The genome contains 4,941 genes, including 4,783 protein-coding genes, 52 tRNAs, and 4 noncoding RNAs. Initial classification with GTDB-Tk v1.7.0 (21) placed GC11 within the genus *Roseomonas* but showed a distant similarity to *Roseomonas cervicalis* ATCC 49957 (GCF_000164635.1). Species identity was then assessed with JSpeciesWS v3.8.2 (22), revealing 83.39% average nucleotide identity (ANI) with *R. cervicalis* ATCC 49957, calculated by ANIdb of the top six genomes selected by Tetra Correlation Search using z-scores. Digital DNA–DNA hybridization (dDDH) d4 values were 29.6% to *R. cervicalis* ATCC 49957 from Type Strain Genome Server (TYGS) v3.3.3 (23), supporting its differentiation from known *Roseomonas*. Functional annotation using DRAM v0.1.0 (24), BlastKOALA v3.1 (25), and PGAP annotation suggests the involvement of *Roseomonas* in complex carbohydrate degradation, nitrite reduction (*nirBD*), and assimilatory sulfate reduction (*cysNC*,* cysD*, *cysH*, and *sir*) in aquatic biochemical processes.

## Data Availability

The data were deposited in the Sequence Read Archive (SRA) under the accession number SRR19987100, BioProject under the accession number PRJNA734631, and BioSample under the accession number SAMN28844363. The whole-genome shotgun projects for *Roseomonas* sp. strain GC11 have been deposited in GenBank under the accession number JANFNY000000000. The version described in this paper is JANFNY000000000.1. The workflow for this genome assembly is within this static KBase narrative DOI: https://doi.org/10.25982/130563.7/2480634. BlastKOALA assignments are available at FigShare and are accessible at DOI: https://doi.org/10.6084/m9.figshare.29586101.
